# Convergent skeletal muscle cytokine responses to TFEB overexpression and voluntary wheel running reflect sex‐based variability to exercise adaptations in mice

**DOI:** 10.14814/phy2.70519

**Published:** 2025-08-20

**Authors:** Dalton C. Patterson, Allison Birnbaum, Ian Matthews, Constanza J. Cortes

**Affiliations:** ^1^ Department of Cell, Developmental and Integrative Biology University of Alabama at Birmingham Birmingham Alabama USA; ^2^ Department of Molecular, Cell and Developmental Biology University of California Los Angeles California USA; ^3^ Leonard Davis School of Gerontology University of Southern California Los Angeles California USA

**Keywords:** cytokines, exercise, running, sex‐differences

## Abstract

Running promotes skeletal muscle remodeling through metabolic and inflammatory signaling pathways, though the extent to which these responses are sex‐dependent remains unclear. We profiled cytokine responses in quadriceps lysates from sedentary, voluntary wheel‐running (VWR), and muscle‐specific TFEB‐overexpressing (cTFEB;HSACre) male and female mice. Cytokine analysis revealed 40 differentially expressed factors associated with exercise and/or TFEB overexpression, many exhibiting sex‐dimorphic patterns. In males, VWR increased interleukins (IL‐1α, IL‐1β, IL‐2, IL‐5, and IL‐17) and chemokines (e.g., MCP‐1, CCL5, and CXCL9), including components of TNF signaling (e.g., TNFα, sTNFR1/2, and Fas ligand). These elevations were partially recapitulated by TFEB overexpression in sedentary males. In contrast, female VWR muscle showed limited changes, with significant differences restricted to IL‐3, IL‐3Rb, IL‐13, and CXCL16. These findings demonstrate sex‐specific cytokine responses to endurance‐like stimuli and suggest a broader or more prolonged inflammatory remodeling profile in male skeletal muscle. Moreover, muscle‐specific TFEB overexpression reproduced similar endurance‐induced cytokine changes, particularly in males, highlighting TFEB as a partial molecular mimic of exercise‐associated inflammatory signaling in skeletal muscle. Together, our data underscore the importance of sex as a biological variable in exercise‐induced cytokine remodeling and support the utility of TFEB overexpression as a platform for prioritizing exercise‐associated phenotypes.

## 1 INTRODUCTION

Cytokines are a diverse family of intracellular signaling molecules regulating multiple organ system functions and maintenance during aging and disease. Cytokines maintain tissue homeostasis through precise signaling between pro‐ and anti‐inflammatory cytokines, and this balance plays an important role in regulating the body's response to exercise (Docherty et al., 2022; MoTrPAC Study Group & Lead Analysts, 2024). There are many sub‐families of cytokines, each with unique functions and effects, such as interleukins (ILs), tumor necrosis factor (TNF), interferons (IFNs), chemokines, colony‐stimulating factors (CSFs), and growth factors. Exercise modulates the expression and secretion of many of these cytokines in skeletal muscle (Gleeson et al., 2011; MoTrPAC Study Group & Lead Analysts, 2024), where they act as autocrine, endocrine, and paracrine mediators (Langston & Mathis, 2024) contributing to the full metabolic and functional adaptations of long‐term exercise (Langston & Mathis, 2024). Indeed, in the context of exercise, the primary source of cytokine responses is the skeletal muscle itself (Docherty et al., 2022). This suggests that cytokines play a central signaling role in the musculoskeletal system and beyond. However, the constellation of exercise‐activated responses on transcriptional, metabolic, and functional outcomes in skeletal muscle is highly complex (MoTrPAC Study Group & Lead Analysts, 2024), with the directionality and intensity of these signatures being highly responsive to variations in experimental cohorts, exercise interventions, and measurement timing and methods (MoTrPAC Study Group & Lead Analysts, 2024), making the isolation of primary effectors from secondary responses difficult (Sanford et al., 2020). Overall, most evidence indicates that after an initial pro‐inflammatory profile following acute exercise (Langston & Mathis, 2024; MoTrPAC Study Group & Lead Analysts, 2024), regular exercise leads to an overall reduction in pro‐inflammatory cytokines and an increase in anti‐inflammatory cytokine profiles (Gleeson et al., 2011; Langston & Mathis, 2024; Prokopchuk et al., 2007), although the degree to which these profiles change remains widely debated. Furthermore, to date, most research into exercise‐activated cytokines has remained male‐centric, and few studies have examined how sex contributes as a biological variable to exercise‐activated cytokine profiles (MoTrPAC Study Group & Lead Analysts, 2024).

Transcription factor E‐B (TFEB) is a master regulator of mitochondrial metabolism and the endolysosomal network (Mansueto et al., 2017; Settembre et al., 2011). The expression and function of TFEB are strongly induced in skeletal muscle in response to exercise (Erlich et al., 2018; Mansueto et al., 2017), and TFEB is required for the full metabolic remodeling observed in skeletal muscle after treadmill training (Mansueto et al., 2017). We have recently demonstrated that overexpression of TFEB using a pan‐skeletal muscle driver (cTFEB;HSACre transgenic mice) is sufficient to drive endurance‐like remodeling of quadriceps, gastrocnemius, and soleus muscle in the absence of any exercise activity (Matthews et al., 2023). Muscle‐TFEB overexpression increased muscle fiber cross‐sectional area, decreased the abundance of fast‐twitch type IIb fibers, and increased the abundance of slow‐twitch type I and IIa fibers in young gastrocnemius muscle (Matthews et al., 2023). We also reported increased exercise endurance capacity in treadmill exhaustion tests for young cTFEB;HSACre transgenic mice of both sexes (Matthews et al., 2023), and preserved quadriceps and gastrocnemius wet weight (skeletal muscle mass) in aged cTFEB;HSACre transgenic mice relative to their age‐matched littermate controls (Matthews et al., 2023). Unbiased proteomic profiling of TFEB‐overexpressing quadriceps muscles revealed multiple categories associated with cellular metabolism and mitochondrial function, including thermogenesis, oxidative phosphorylation, fatty acid metabolism, and amino acid metabolism (Matthews et al., 2023). This is consistent with how acute AAV‐mediated TFEB expression in young male skeletal muscle was previously shown to induce the expression of genes involved in mitochondrial biogenesis and oxidative phosphorylation, increasing exercise endurance via improving glycogen metabolism (Mansueto et al., 2017). We also demonstrated that muscle‐TFEB overexpression promotes the secretion of exercise‐responsive, muscle‐originating factors into circulation (i.e., exerkines), including cathepsin B (Matthews et al., 2023) and prosaposin (Taha et al., 2024). Importantly, we were also the first to evaluate these local and systemic exercise‐like effects across both sexes and identified both sex‐ and age‐dimorphic trajectories associated with TFEB‐overexpression in skeletal muscle (Matthews et al., 2023). Altogether, this suggests that muscle‐TFEB overexpression promotes maintenance of mitochondrial function and bioenergetic reserves, induces running‐like fiber type switching, and preserves skeletal muscle function during aging, all profiles reminiscent of those seen after endurance training (Cartee et al., 2016). Based on this prior evidence, we hypothesized that muscle‐specific overexpression of TFEB across both sexes would also recapitulate aspects of cytokine profiles associated with endurance exercise. Here, we report that cytokine signatures from cTFEB;HSACre skeletal muscle partially overlap with cytokine signatures from endurance exercised muscle, suggesting it represents an intermediate profile between sedentary and exercised states. Furthermore, we find that female endurance exercised skeletal muscle cytokine profiles do not follow the same patterns as their male counterparts, but they are reminiscent of female muscle‐TFEB overexpressing muscle, highlighting conserved sex‐dimorphic responses to exercise and TFEB‐mediated physiology. Altogether, we present evidence highlighting the potential of cTFEB;HSACre transgenic mice as a powerful orthogonal tool for examining and prioritizing skeletal muscle exercise‐associated profiles (Matthews et al., 2023; Taha et al., 2024).

## 2 METHODS

### 2.1 Animals

**FIGURE 1:**
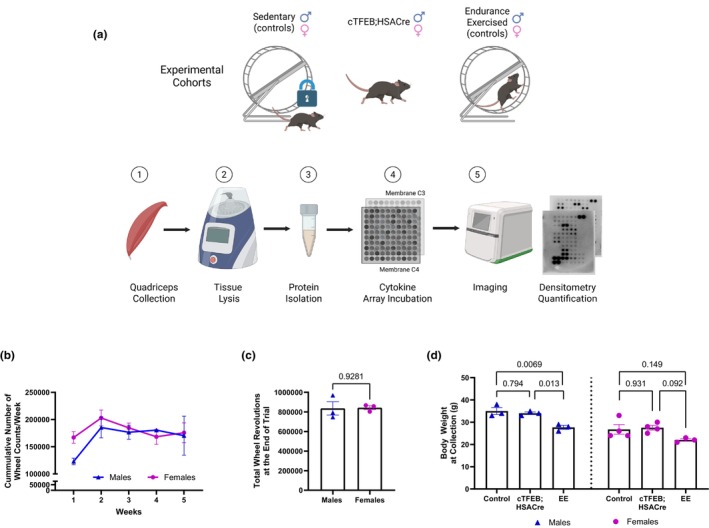
Voluntary running wheel (VRW) performance and body weight in experimental cohorts. (a) Design schematic highlighting experimental cohorts: Sedentary (*n* = 4 M/F), cTFEB;HSACre transgenic (*n* = 4 M/F) and endurance exercised (EE) (*n* = 3 M/F) mice, 4.5 months of age at the beginning of the experiment. Sample processing and analysis pipeline is shown below. (b) No significant differences in cumulative wheel counts/week throughout the course of the experiment. (c) Total wheel counts at the end of the experiment for both sexes. (d) Significantly reduced body weight in endurance exercised (EE) males (and trending for females) at collection; no differences in cTFEB;HSACre transgenic mice body weight relative to sedentary controls. *p* values as shown. Data is represented as mean ± SEM.

We have previously described the generation of fxSTOP‐TFEB transgenic and cTFEB;HSACre double transgenic mice (Mansueto et al., 2017). For this work, we utilized controls or cTFEB;HSACre double transgenic male and female littermates (~4–5 months of age, *n* = 4/sex). In our hands, we have not detected any differences in muscle phenotypes on fxSTOP‐TFEB+ or HSA‐Cre + single transgenic animals compared to wild‐type littermates (Mansueto et al., 2017), so they are all combined into our “control” groups. Control animals were randomly assigned to each sedentary (*n* = 4/sex) or runner (*n* = 3/sex) group (see below). We utilized similar numbers of males and females. Body weight was measured prior to euthanasia and tissue collection and was accidentally not collected for one male control and one male cTFEB;HSACre animal (see Figure 1d). All animals are in the C57BL/6J genetic background and are maintained in standard shoebox‐style cages with filter tops with a 12:12 light–dark cycle. Mice were not fasted before tissue collection, and they were allowed free access to standard NIH‐07 mouse chow throughout the experiment. All animal experimentation adhered to NIH guidelines and was performed by blinded investigators (when possible) and was approved by and performed in accordance with the University of Alabama at Birmingham (UAB) and the University of Southern California (USC) Institutional Animal Care and Use.

### 2.2 Exercise protocol

Four‐month‐old control mice (*n* = 3) were singly housed with access to in‐cage locked (sedentary) or free (running) wheels for 5 weeks at the UAB Small Animal Phenotyping Core. To equilibrate the influence of single housing on animal behavior, each mouse from sedentary/runner cohorts was single‐caged in standard vivarium polypropylene cages (290 × 180 × 160 mm) throughout the experiment in an incubator that maintained a constant temperature. Each running mouse had free access to a running wheel (13 cm in diameter) connected to a counter to record the running distance (Lafayette Science's AWM activity monitoring software). This in‐cage wheel running system for mice collects the number of revolutions and the timing of the revolutions for each cage every 10 min. Sedentary controls were also given free access to a locked wheel to control for enrichment. Investigators monitored animal welfare and physical activity daily.

### 2.3 Tissue collection

Tissue collections were all undertaken between 9 and 11 am on the last day of the fifth week of the trial. Animals were anesthetized with 3.8% Avertin Solution prior to tissue collection. Euthanasia was completed via diaphragm transection followed by transcardial perfusion with 60 mL of ice‐cold 1x PBS. Quadriceps skeletal muscle tissue was flash‐frozen in liquid nitrogen for protein extraction and downstream analyses.

### 2.4 Protein extraction

Protein lysates from muscle tissue were prepared following standard protocols with minor modifications (Cortes, Ling, et al., 2014; Cortes, Miranda, et al., 2014; Matthews et al., 2023). In short, 50 mg of flash‐frozen perfused quadriceps muscle were placed in plastic tubes with silica beads (MP Biomedical,116913100) and homogenized in the FastPrep‐24 5G bead grinder and lysis system (MP Biomedical, 116005500) in RIPA Lysis and Extraction Buffer (Invitrogen, 89900), 1X Halt Protease and Phosphatase Inhibitor Cocktail (Invitrogen, 78442), and 1% SDS. After standard centrifugation steps, protein concentration was quantified using a Pierce BCA Protein Assay (23227).

### 2.5 Cytokine Array

Cytokine antibody arrays (Abcam, ab193659) were used according to the manufacturer's protocol. For skeletal muscle analysis, 50 μg of previously prepared quadriceps protein lysate was incubated with the membranes precoated with captured antibodies (C3: 62 targets and membrane C4: 34 targets). Targets analyzed were as follows: Axl, Bfgf, BLC (CXCL13), CD30 Ligand (TNFSF8), CD30 (TNFRSF8), CD40 (TNFRSF5), CRG‐2, CTACK (CCL27), CXCL16, CD26 (DPPIV), Dtk, Eotaxin‐1 (CCL11), Eotaxin‐2 (MPIF‐2/CCL24), E‐Selectin, Fas Ligand (TNFSF6), Fc gamma RIIB (CD32b), Flt‐3 Ligand, Fractalkine (CX3CL1), GCSF, GITR (TNFRSF18), GM‐CSF, HGFR, ICAM‐1 (CD54) IFN‐gamma, IGFBP‐2, IGFBP‐3, IGFBP‐5, IGFBP‐6, IGF‐1, IGF‐2, IL‐1 beta (IL‐1 F2), IL‐10, IL‐12 p40/p70, IL‐12 p70, IL‐13, IL‐15, IL‐17A, IL‐17 RB, IL‐1 alpha (IL‐1 F1), IL‐2, IL‐3, IL‐3 R beta, IL‐4, IL‐5, IL‐6, IL‐7, IL‐9, I‐TAC (CXCL11), KC (CXCL1), Leptin, Leptin R, LIX, L‐Selectin (CD62L), Lungkine (CXCL15), Lymphotactin (XCL1), MCP‐1 (CCL2), MCP‐5, M‐CSF, MDC (CCL22), MIG (CXCL9), MIP‐1 alpha (CCL3), MIP‐1 gamma, MIP‐2, MIP‐3 beta (CCL19), MIP‐3 alpha (CCL20), MMP‐2, MMP‐3, Osteopontin (SPP1), Osteoprotegerin (TNFRSF11B), Platelet Factor 4 (CXCL4), Pro‐MMP‐9, P‐Selectin, RANTES (CCL5), Resistin, SCF, SDF‐1 alpha (CXCL12 alpha), Sonic Hedgehog N‐Terminal (Shh‐N), TNF RI (TNFRSF1A), TNF RII (TNFRSF1B), TARC (CCL17), I‐309 (TCA‐3/CCL1), TECK (CCL25), TCK‐1 (CXCL7), TIMP‐1, TIMP‐2, TNF alpha, Thrombopoietin (TPO), TRANCE (TNFSF11), TROY (TNFRSF19), TSLP, VCAM‐1 (CD106), VEGF‐A, VEGFR1, VEGFR2, VEGFR3, and VEGF‐D. After washing, the membranes were incubated with the provided streptavidin‐HRP secondary detection antibodies. Membranes were developed using the included chemiluminescent‐based Detection Buffers C and D. Array blot images were captured and visualized using the ChemiDoc MP Imaging System (Bio‐Rad), and the intensity of each spot (densitometry) in the captured images was analyzed using the publicly available software ImageJ as per kit instructions. To determine fold changes associated with cTFEB;HSACre or running status, relative cytokine levels were all normalized to sedentary control values for each sex. Results are expressed as arbitrary units/fold change over sedentary controls for each sex. Only cytokines with significant differences are shown.

### 2.6 Statistical analysis

Given small sample sizes, all data were analyzed by one‐way ANOVA within sex with post hoc comparisons using GraphPad Prism 10.4.2 (La Jolla, CA) and are represented as means and standard error of the means. All data analyses were conducted in a blinded fashion. All data were prepared for analysis with standard spreadsheet software (Microsoft Excel).

## 3 RESULTS

### 3.1 Running‐activated cytokine profiles are sex‐dimorphic

To profile the shared and unique cytokine responses associated with endurance‐like muscle remodeling in male and female mice, we generated quadriceps muscle protein lysates from young (4.5 months old) sedentary, exercised (voluntary wheel running, VWR) or cTFEB;HSACre transgenic mice of both sexes (*n* = 3–4/group) (Figure 1a). We selected quadriceps muscle as our tissue of interest as it was the main skeletal muscle type we characterized in our original report describing the effects of TFEB overexpression in cTFEB;HSACre transgenic mice (Matthews et al., 2023), it is known to be remodeled via VWR (Hamm et al., 2023; Sexton, 1995; Soffe et al., 2016), and displays classical hallmarks of skeletal muscle aging that are responsive to endurance training, including increases in inflammation and decreased mitochondrial function (Cartee et al., 2016). Mice in the running group were provided 24‐h access to an in‐cage running wheel for 5 weeks, whereas the sedentary group remained in cages with a locked wheel. This length of running intervention has been previously shown to be sufficient to promote exercise‐associated muscle adaptations, including increased mitochondrial activity (Ikeda et al., 2006; LaBarge et al., 2016), increased capillary density (Waters et al., 2004), and fiber type switching (Allen et al., 2001; Waters et al., 2004) in young mice. We chose this voluntary running paradigm to avoid stress‐induced activation of inflammation associated with long‐term forced treadmill running (Cook et al., 2013; Svensson et al., 2016). Consistent with previous reports monitoring similar durations of voluntary running in wild‐type mice at similar ages (Bartling et al., 2017), we observed continuous running behavior across 5 weeks for both male and female mice (Figure 1b). Interestingly, female mice displayed a higher preference for running over their male littermate counterparts during the wheel acclimation period (Figure 1b, week 1). Male runners caught up to female runners in both the average (not shown) and cumulative wheel counts by week 3 and remained comparable till the end of the experiment (Figure 1b, weeks 2–5). Indeed, males and females had completed similar total wheel counts by the end of the study (Figure 1c). Body weight measurements at the end of the running trials confirmed that 5 weeks of VWR reduced total body weight in male (and trending for female) runners (Figure 1d), consistent with the well‐known benefits of VWR on fat/lean mass (Bartling et al., 2017). As we have shown previously (Matthews et al., 2023), no differences in body weight were observed in young male/female cTFEB;HSACre transgenic mice when compared to their littermate controls (Figure 1d).

Next, we simultaneously assessed 96 mouse inflammation and immune system responsive cytokines in quadriceps muscle total protein lysates from all groups (Figure 1a). Of these, we identified a total of 40 differentially expressed cytokines, some of which were uniquely associated with running (17), with muscle‐TFEB overexpression (4), or following similar directional changes under both conditions (19). Notably, most of these also exhibited a sex‐dimorphic pattern, with robust exercise and cTFEB;HSACre signatures in males that were absent in females. We describe our findings below.

#### 3.1.1 Interleukins (ILs)

**FIGURE 2:**
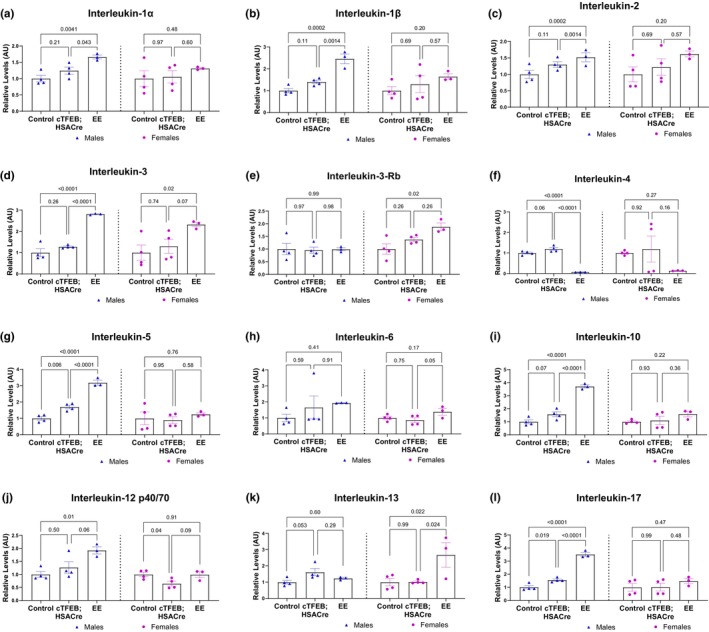
Interleukin cytokine profiles of cTFEB;HSACre and endurance exercised skeletal muscle lysates. (a–l) Densitometry analysis of interleukin abundance in skeletal muscle protein lysates of all cohorts normalized to respective sedentary baseline for each sex. Arbitrary units. *p* values as shown. Data is represented as mean ± SEM.

Interleukins (ILs) are a group of proteins involved in communication with leukocytes by binding to specific receptors. ILs are mainly synthesized by monocytes, macrophages, T cells, and endothelial cells, but evidence suggests myofibers and myoblasts can also produce ILs (Peake, Della Gatta, et al., 2015). ILs are highly responsive to both the timing and intensity of the exercise bout, with differential effects on the pro‐inflammatory and anti‐inflammatory branches of the family (Gleeson et al., 2011; Peake, Della Gatta, et al., 2015; Prokopchuk et al., 2007). In our hands, and similar to what has been previously shown (Peake, Della Gatta, et al., 2015), we detected significant increases in pro‐inflammatory cytokines IL‐1α, IL‐1β, IL‐2, IL‐3, IL‐5, IL‐12 p40/70, IL‐12 P70 (not shown), and IL‐17 in skeletal muscle lysates from male young endurance exercised mice (Figure 2). We did not detect any changes in IL‐6 levels in endurance exercised muscle lysates of either sex (Figure 2h), consistent with its role as an early responder to acute bouts of endurance exercise. Interestingly, although many of these cytokines appeared to trend in the same direction as their male counterparts, only IL‐3 was also significantly elevated in the muscle of female endurance exercisers (Figure 2d). Furthermore, pro‐inflammatory cytokine IL‐3Rb (Figure 2e) was found to be significantly increased in female (but not male) muscle lysates after 5 weeks of voluntary wheel running. Running also robustly decreased the abundance of anti‐inflammatory cytokine IL‐4 in both sexes (Figure 2f) and increased levels of anti‐inflammatory IL‐10 (males only) (Figure 2i) and IL‐13 (females only) (Figure 2k). Interestingly, out of these, both IL‐5 (Figure 2g) and IL‐17 (Figure 2l) were also significantly upregulated in the muscle lysates of age‐matched male (but not female) cTFEB;HSACre transgenic mice, even in the absence of running.

#### 3.1.2 Chemokines

**FIGURE 3:**
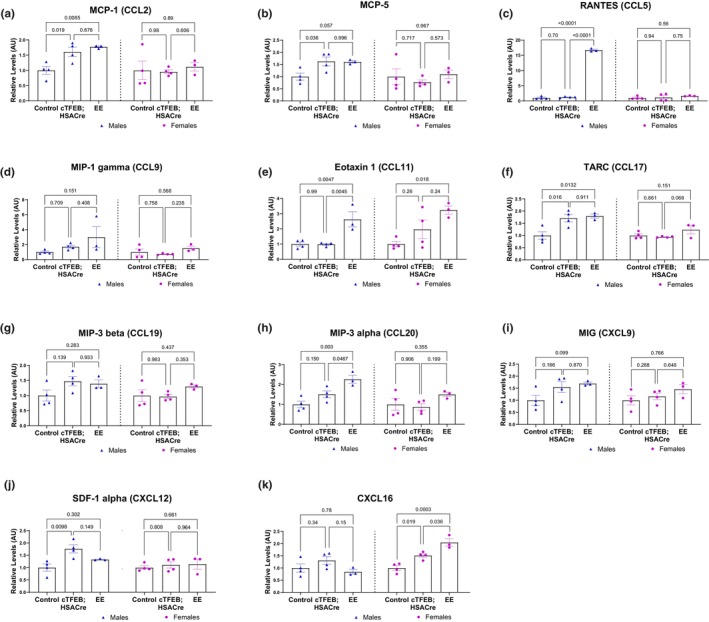
Chemokine cytokine profiles of cTFEB;HSACre and endurance exercised skeletal muscle lysates. (a–k) Densitometry analysis of chemokine abundance in skeletal muscle protein lysates of all cohorts normalized to respective sedentary baseline for each sex. Arbitrary units. *p* values as shown. Data is represented as mean ± SEM.

Chemokines are another large family of exercise‐responsive cytokines (Docherty et al., 2022; Gleeson et al., 2011; Peake, Della Gatta, et al., 2015), which function to stimulate the recruitment of leukocytes and other immune cells from circulation into solid tissues (chemotaxis). They can be divided into two families, with conserved sequential (CC) or proximal (CXC) cysteine residues. Examination using our ELISA approach revealed nine differentially expressed chemokines in skeletal muscle lysates from male young endurance exercised mice (Figure 3), including MCP‐1/CCL2, MCP‐5, RANTES/CCL5, Eotaxin 1/CCL11, TARC/CCL17, MIP‐3 alpha/CCL20, and MIG/CXCL9. Similar to what we observed in our interleukin panel, these chemokine levels did not change in young female endurance exercised skeletal muscle relative to their baseline sedentary controls (Figure 3), although we detected an additional significant increase in CXCL16 levels in female (but not male) endurance exercised muscle (Figure 3k). TFEB‐overexpressing muscle also displayed significant increases in MCP‐1 (CCL2) (Figure 3a) and TARC (CCL17) (Figure 3f) to similar levels as their endurance exercised littermates, and CXCL16 to a level in between sedentary and endurance exercised littermates (Figure 3k), following the same sex‐specific patterns that we saw in endurance exercised chemokine profiles (Figure 3). TFEB‐overexpressing muscle lysates displayed one unique significant increase in CXCL family member SDF‐1 alpha/CXCL12 relative to their non‐transgenic sedentary control littermates (Figure 3j).

#### 3.1.3 Tumor necrosis factor (TNF) signaling

**FIGURE 4:**
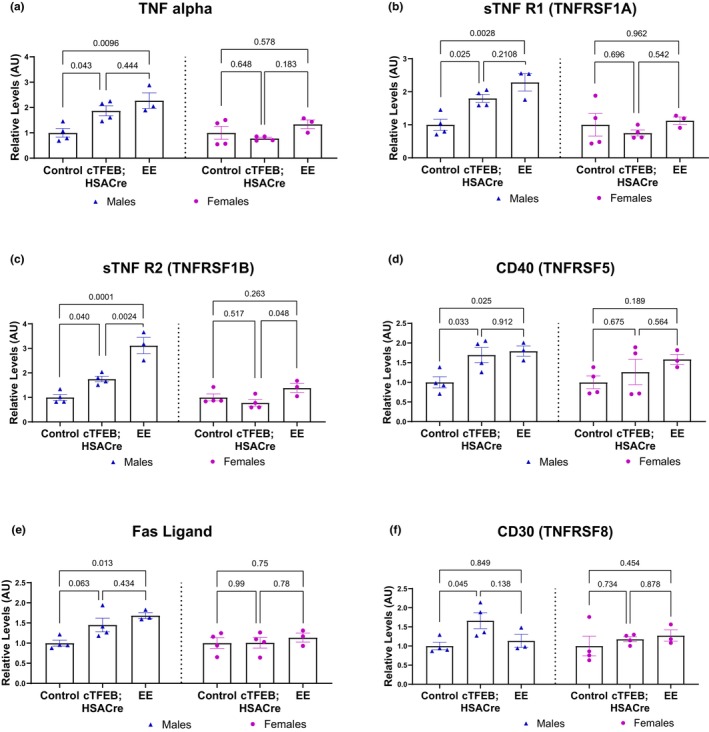
TNF signaling cytokine profiles of cTFEB;HSACre and endurance exercised skeletal muscle lysates. (a–f) Densitometry analysis of TNF cytokine family abundance in skeletal muscle protein lysates of all cohorts normalized to respective sedentary baseline for each sex. Arbitrary units. *p* values as shown. Data is represented as mean ± SEM.

Tumor necrosis factor alpha (TNF‐α) is a pluripotent cytokine generally produced by macrophages and adipocytes and is a major mediator of the acute inflammatory response (Sethi & Hotamisligil, 2021). TNF‐α has two membrane‐bound receptors that can be cleaved into soluble isoforms involved in its signaling cascade and likely modulate its dual effects on inflammation: sTNFR1 is constitutively expressed on most cell types, and its signaling is mainly pro‐inflammatory, whereas sTNFR2 is primarily restricted to subsets of immune cells, glial cells, and cardiomyocytes, and its signaling can be anti‐inflammatory and promote cell proliferation instead (Sethi & Hotamisligil, 2021). TNF levels increase with endurance exercise, consistent with what we found in this study (Figure 4). Male endurance exercised muscle had significantly elevated levels of cytokines involved in the TNF signaling axis, including TNFα, soluble TNFR1, soluble TNFR2, CD40/TNFRSF5, and Fas ligand (Figure 4a–e). We found that male cTFEB;HSACre muscle also displayed significant elevations in these cytokines compared to their sedentary littermate controls, to a degree indistinguishable from endurance exercised muscle (Figure 4a–e). Furthermore, cTFEB;HSACre muscle also had significantly higher cytokine CD30/TNFRSF8 levels (Figure 4f), another member of the TNF signaling axis. Once again, no changes were detected for any TNF‐associated cytokines in the skeletal muscle of female cTFEB;HSACre or endurance exercised mice (Figure 4).

#### 3.1.4 Additional cytokines

**FIGURE 5:**
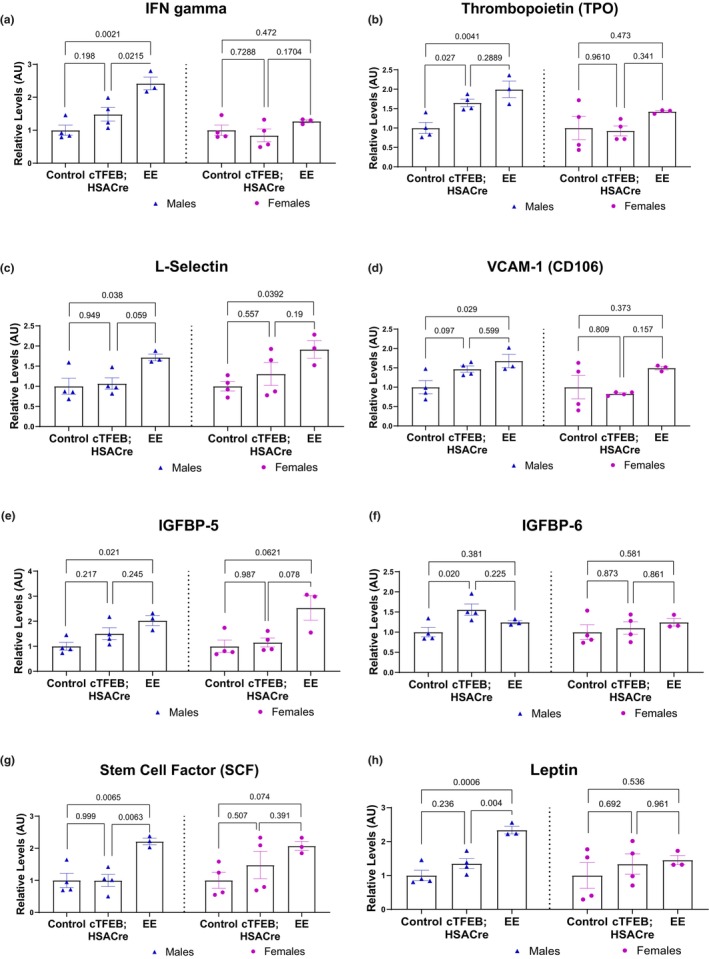
Orphan signaling cytokine profiles of cTFEB;HSACre and endurance exercised skeletal muscle lysates. (a–h) Densitometry analysis of additional cytokine family abundance in skeletal muscle protein lysates of all cohorts normalized to respective sedentary baseline for each sex. Arbitrary units. *p* values as shown. Data is represented as mean ± SEM.

Interferon‐gamma (IFNγ) increases in human plasma after regular moderate exercise (Vijayaraghava & Radhika, 2014) and immediately after acute moderate exercise (Viti et al., 1985) and has been linked to the anti‐inflammatory benefits of regular physical activity. We detected a significant increase in IFNγ abundance in muscle lysates from male endurance exercised and cTFEB;HSACre transgenic muscle, although to a lesser degree (Figure 5a). No changes were detected for IFNγ levels in the muscle of female mice of either group (Figure 5a).

Thrombopoietin (TPO), a glycoprotein hormone that regulates platelet production and may improve osteogenesis in the absence of mechanical loading (Zamarioli et al., 2021), was also significantly elevated in both male endurance exercised and male cTFEB;HSACre muscle (Figure 5b). L‐selectin and Vascular cell adhesion molecule 1 A (VCAM1A) are cell adhesion molecules that play a role in inflammation responses and leukocyte adhesion, potentially modulating immune cell infiltration during inflammation. We found increases in both of these cytokines in the muscle of our male (and trending for female) endurance exercised groups (Figure 5c,d).

IGFBP‐5, an insulin‐like growth factor binding protein that stimulates IGF‐1 signaling, plays a complex role in skeletal muscle, influencing both muscle growth and atrophy (Awede et al., 1999), and appears to decrease in the interstitial muscle space in human muscle after acute exercise (Nindl et al., 2020). Importantly, IGFBPs are known to have IGF‐independent actions in cell migration, cell growth, and apoptosis (Mohan & Baylink, 2002). We found a significant increase in IGFBP‐5 levels in both male and female endurance exercised muscle relative to their sedentary littermate controls (Figure 5e). IGFBP‐6, another insulin‐like growth factor binding protein family member that inhibits IGF‐1 signaling, is increased in the muscle of mice selected for high running performance (Brenmoehl et al., 2021) and in regenerating skeletal muscle (Yamaguchi et al., 2013). We found a significant increase in abundance levels of IGFBP‐6 in male cTFEB;HSACre (but not endurance exercised) muscle (Figure 5f).

Stem cell factor (SCF) is a cytokine that recruits stem cells from the bone marrow into sites of injury via activation of receptor tyrosine kinase c‐Kit (Lennartsson & Ronnstrand, 2012). SCF was also found to be significantly elevated in male endurance exercised (but not cTFEB;HSACre) muscle, with similar trends in female endurance exercised muscle (Figure 5g). Finally, we also observed significantly increased leptin levels, a hormone commonly synthesized by fat tissue, which has been noted to regulate energy balance and metabolism (Bouassida et al., 2006), in male endurance exercised muscle lysates (Figure 5h).

## 4 DISCUSSION

Exercise alters cytokine production in skeletal muscle (Docherty et al., 2022; Gleeson et al., 2011; MoTrPAC Study Group & Lead Analysts, 2024; Peake, Della Gatta, et al., 2015), but the role biological sex plays in these cytokine profiles remains largely unknown. Here, we provide evidence that cytokine profiles derived from quadriceps skeletal muscle lysates following 5 weeks of VWR differ significantly between female and male mice, demonstrate that muscle‐TFEB overexpression mimics aspects of this sex‐dimorphic cytokine response to voluntary wheel running in mice, and discuss the potential contributions of muscle resident and infiltrating cell types to these profiles.

Most exercise‐regulated cytokines examined here play myriad roles in the immune and nonimmune systems (Gleeson et al., 2011). Indeed, pro‐ and anti‐inflammatory cytokines are altered by acute and chronic exercise in rodents and humans (Pedersen & Hoffman‐Goetz, 2000), although their physiological effects are context‐, timing‐, and cell‐type specific (Langston & Mathis, 2024). It has been suggested that the local beneficial impact of chronic exercise is mediated, at least in part, by exercise‐induced “hormesis” (Peake, Markworth, et al., 2015), whereby a low level of stress (in this case, associated with the increased metabolic demand of the running bout) upregulates existing cellular and molecular pathways that improve physiological hallmarks (Langston & Mathis, 2024). Thus, specific combinations of cytokines (sometimes with opposing functions) and the directionality, magnitude, and duration of these changes likely underlie metabolic and architectural remodeling of skeletal muscle in response to running. Consistent with this hypothesis, we identified multiple inflammation‐associated cytokines (ILs, TNF signaling, chemokines) that are significantly elevated in male skeletal muscle after 5 weeks of VWR. Many of these have been reported to be exercise‐responsive before, both within skeletal muscle (Little et al., 2018) and in circulation (Little et al., 2018; Peake, Della Gatta, et al., 2015). In fact, studies collectively demonstrate that 5–8 weeks of other endurance training methods, including treadmill training or ladder‐climbing, modify levels of pro‐inflammatory cytokines (IL‐1β, IL‐6, and TNF‐α) and anti‐inflammatory factors (IL‐10) in rodent hindlimb muscles such as the EDL, soleus, and gastrocnemius (Calegari et al., 2018; Isanejad et al., 2015; Lira et al., 2009). This supports a coordinated remodeling of the muscle cytokine environment associated with medium‐term exercise interventions. Interestingly, 1 week of VWR has been shown to induce more cytokine changes than either an acute low‐intensity or high‐intensity exercise in mice (Little et al., 2018), further highlighting the importance of timing and intervention versus recovery phases during exercise studies. The fact that pro‐inflammatory cytokines remain elevated in our 5‐week VWR intervention suggests that these cytokine profiles may be associated with ongoing exercise‐dependent muscle remodeling rather than muscle damage, although this hypothesis remains to be tested.

Many of the differentially expressed cytokines we identified in this study are chemoattractants (chemokines), adhesion molecules that facilitate extravasation (VCAM1 and L‐Selectin), or immune‐system signaling molecules (interleukins). Skeletal muscle has both resident and exercise‐responsive immunocyte populations, including macrophages, T‐regs, and neutrophils, and exercise has well‐known effects on immune cell circulation and tissue‐resident populations (Gleeson et al., 2011; Langston & Mathis, 2024; Pedersen & Hoffman‐Goetz, 2000). Importantly, the fold changes we observed in most canonically known pro‐inflammatory interleukins (IL‐1α, IL‐1β, IL‐2, IL‐5, IL‐12 p40/70, and IL‐17) are mild (between 2‐ and 4‐fold over sedentary controls) and may reflect tissue‐remodeling responses or chronic sub‐pathological levels of inflammation (“inflammation hormesis”). Additionally, the chemokine profiles we detected suggest a local increase in the production of chemoattractants targeting monocytes (MCP‐1/CCL2, MCP‐5), macrophages (MIP‐1 gamma/CCL9), T‐cells (MIP‐3 beta/CCL19, MIP‐3 alpha/CCL20, MIG/CXCL9, and TARC/CCL17), eosinophils (Eotaxin 1/CCL11), and leukocytes (RANTES/CCL5). It is currently unclear whether these profiles reflect an increase in local immunocyte signaling or an exercise‐dependent infiltration of new immune cells into endurance exercised and/or cTFEB;HSACre muscle. Although we cannot rule out this possibility, the type and duration of exercise the mice were subjected to in our study generally do not cause significant muscle damage (Little et al., 2018), suggesting that if these are new infiltrating immunocytes, they may be involved in tissue remodeling rather than tissue repair responses.

We (Matthews et al., 2023), and others (Mansueto et al., 2017; Matthews et al., 2023), have previously shown that muscle‐TFEB overexpression engages multiple exercise‐like effects in skeletal muscle, including mitochondrial remodeling, fiber type switching, and expansion of the endolysosomal network. Unbiased proteomics analysis identified many classical exercise‐activated networks in TFEB‐overexpressing quadriceps muscle, including proteostasis, mitochondrial metabolism, fatty acid metabolism, and amino acid metabolism (Matthews et al., 2023). Furthermore, we also confirmed that muscle‐TFEB overexpression increases the secretion of exercise‐responsive factors into circulation (Matthews et al., 2023), which promotes exercise‐like remodeling in the Central Nervous System (Matthews et al., 2023; Taha et al., 2024). Here, we expand our original analysis of the effects of TFEB‐overexpression in skeletal muscle and demonstrate that it also mimics cytokine profiles associated with voluntary wheel running. Indeed, we detected significantly increased levels of interleukins (IL‐5 and IL‐17), chemokines (MCP‐1/CCL2, TARC/CCL‐17, and CXCL16), and TNF signaling (TNFα, sTNFR1, sTNFRII, and CD40/TNFRSF5) in cTFEB;HSACre skeletal muscle to levels similar (or intermediate) to those seen in endurance‐exercised muscle.

Interestingly, the strongest concordance in cytokine profiles between cTFEB;HSACre and endurance exercised muscle was observed in the TNF signaling pathway. Although commonly associated with muscle tissue loss in conditions of cachexia or sarcopenia, TNF‐α also has important roles in muscle repair (Warren et al., 2002) and growth and differentiation (Li, 2003; MacLachlan & Giordano, 1998), and can be produced locally by myocytes and myoblasts (Peake, Della Gatta, et al., 2015; Saghizadeh et al., 1996). The increases we observed in TNF signaling cytokines in either cTFEB;HSACre or endurance exercised lysates are much lower than those reported for muscle pathological conditions, suggesting that they may instead represent immunomodulatory states associated with these anabolic functions of TNF signaling. In agreement with this, in cTFEB;HSACre muscle lysates we observed significant increases in sTNFRII, which is considered mostly anti‐inflammatory and promotes cell proliferation (Sethi & Hotamisligil, 2021). Interestingly, TNFα also induces the surface expression of various adhesion molecules to promote the migration of leukocytes at sites of inflammation (including vascular cell adhesion molecule‐1 (VCAM‐1) and L/E‐selectin), which we also found were significantly increased in endurance exercised muscle lysates. As before, whether these increases reflect local production of TNF‐signaling cytokines or an increased infiltration of immunocytes into either cTFEB;HSACre or endurance exercised muscle remains unknown.

An interesting finding in our study was the absence of significant elevations in IL‐6 in endurance‐exercised or cTFEB;HSACre muscle lysates of either sex. IL‐6 has long been established as an exercise‐responsive cytokine, with levels of IL‐6 sharply increasing in circulation soon after the exercise bout (Docherty et al., 2022; Little et al., 2018; Peake, Della Gatta, et al., 2015). However, much less is known about its levels in skeletal muscle tissue. Some studies suggest that chronic exercise decreases cytokine production (including IL‐6) in rat skeletal muscle (Lira et al., 2009), and that endurance training markedly reduces exercise‐induced IL‐6 mRNA expression in human skeletal muscle (Fischer et al., 2004), potentially reflecting underlying metabolic remodeling processes that blunt IL‐6 activation (Knudsen et al., 2017). Our findings thus likely reflect the fact that 5 weeks of VWR elicited a metabolic adaptation in skeletal muscle sufficient to diminish the reliance on IL‐6 induction as a regulatory signal.

One of our most unexpected findings was the profound sex bias exhibited by almost all the cytokines in this panel. Out of 40 differentially abundant cytokines profiled in our study, only two followed the same patterns in male and female endurance exercised muscle (IL‐3 and IL‐4). Furthermore, three cytokines appeared to be female‐specific (IL‐3Rb, IL‐13, and CXCL16), with significant increases in female (but not male) endurance exercised muscle lysates. It is important to note that we have confirmed human TFEB transgenic protein expression levels to be 3‐fold higher in male than female cTFEB;HSACre skeletal muscle (Matthews et al., 2023). While this may account for some of the sex‐specific changes in cytokines we report here, the fact that the cytokine abundance pattern of female endurance exercised muscle was similar to what we observed in female cTFEB;HSACre muscle instead suggests that conserved sex‐dimorphic signals may control cytokine abundance across both models.

Sex differences in exercise responses remain a profoundly understudied topic of research (Cortes & De Miguel, 2022; MoTrPAC Study Group & Lead Analysts, 2024), and in general, we struggled to identify previous literature describing female‐specific changes to many of these cytokines in response to any exercise modality. This renders interpretation of these female‐specific patterns (or lack thereof) difficult, but we believe a set of overlapping and/or non‐mutually exclusive scenarios could be at play here. In our hands, both sexes displayed similar running patterns (total wheel revolutions at the end of the 5 weeks of VWR) and similar trends towards decreases in body weight between our sedentary and endurance exercised groups of both sexes. This suggests that the intensity of the running intervention had similar systemic effects on the fat/lean mass of our groups, although specific biological responses in adipose tissue, liver, and other highly metabolic tissues were not examined in this work. Another option is the timing of the cytokine responses: our analysis was performed at the end of 5 weeks of running. We did observe a significantly higher number of cumulative wheel counts during Weeks 1–2 in our female runners relative to their male littermates. This may indicate endurance exercised females may reach peak cytokine increases earlier than their male counterparts, with compensatory responses returning these profiles to baseline by the time we collected our samples. Finally, it could also be that female and male skeletal muscle possess different steady‐state profiles for these cytokines, which makes exercise or muscle‐TFEB‐activated changes different across sexes, although we did not examine this possibility in this current work.

Both sexes display the well‐known benefits of exercise on skeletal muscle, suggesting converging metabolic and functional outcomes despite differences in timing, abundance, or cross talk between these immune‐associated cytokines. Thus, it may be that a specific combination of cytokines (with diverse and opposing functions), whose direction, magnitude, and duration of change also appear to be highly sex‐dimorphic, underlie exercise‐induced hormesis. In agreement with this, the recently released Molecular Transducers of Physical Activity Consortium (MoTrPAC) dataset has also highlighted the diverse temporal and sex dynamics of multi‐omic responses to endurance training in young rats (MoTrPAC Study Group & Lead Analysts, 2024). Targeted interrogation of the MoTrPAC data hub for transcriptional and proteomic levels of detected cytokines also present in the cytokine array utilized in this study reveals trends towards sex‐dimorphic responses for IL‐1α, IL‐2, IL‐12 p40/70, IL‐13, and IFN‐γ. Furthermore, the temporal responses of IL‐1α and IL‐12, for example, are also increased during week 1 and disappear by week 4 of the treadmill training in the gastrocnemius of female endurance exercised rats (MoTrPAC Study Group & Lead Analysts, 2024), consistent with our hypothesis that biological sex may play an important role in exercise‐activated cytokine timing and abundance. Together, these studies underscore the fundamental need to examine sex differences that contribute to exercise physiology.

One major limitation of our work was the small sample sizes we utilized (*n* = 3–4/group). Despite this limitation, we were able to identify a large number of differentially abundant cytokines in skeletal muscle lysates from endurance‐trained and/or cTFEB;HSACre mice, which, as mentioned above, reflect similar patterns identified by larger comprehensive studies with high biological and statistical power (i.e., MoTrPAC). This suggests that multiple other cytokines, which displayed trends but did not reach statistical significance in our study, might also be currently understudied exercise‐associated responses, a potential future direction for the work we present here.

Cytokine networks and their responses to exercise signaling must be tightly regulated to limit damage while maintaining the benefits, some of which appear to be recapitulated in TFEB‐overexpressing muscle. Overall, the similarities we observed in our cytokine profiling of endurance exercised and cTFEB;HSACre skeletal muscle suggest that muscle‐TFEB overexpression may prime skeletal muscle to an intermediate exercise‐like state. Indeed, we see cTFEB;HSACre skeletal muscle adopting both characteristics of the initial phase of the immune response during exercise (Langston & Mathis, 2024) (with pro‐inflammatory states characterized by cytokines such as IL‐5, IL‐17, and TNFα) but also of the later phases of the exercise‐activated immune response (with a preponderance of anti‐inflammatory states, characterized by IL‐10, IL‐13, and sTNFR2) (Langston & Mathis, 2024). Furthermore, for several chemokines that did not reach statistical significance (MCP‐5, MIP‐3 alpha, MIG, and leptin, for example), muscle‐TFEB overexpressing samples displayed clear trends towards similar increases as those seen in our endurance exercised groups, suggesting additional nodes of shared signaling between these two models. This highlights the potential of the cTFEB;HSACre transgenic mouse model as a new orthogonal prioritization platform to identify and validate exercise‐associated signaling, including cytokines, secreted factors (Mansueto et al., 2017; Matthews et al., 2023), and age‐associated geroprotective benefits.

## AUTHOR CONTRIBUTIONS

D.C.P., A.B., and I.M. performed the experimental work and reviewed the manuscript. D.C.P. and C.J.C. wrote and edited the manuscript. C.J.C. conceptualized the experiments outlined here and obtained (or helped obtain) all associated funding.

## FUNDING INFORMATION

This work was supported by NIH/NIA Administrative Supplement 3RF1AG057264—03W1 (to D.P. by C.JC.), NIA T32 AG052374 (training grant to I.M. and A.B.), AARF‐21–851362 (to A.B), and NIH/NIA R01 AG077536 (to C.J.C.).

## ETHICS STATEMENT

This work is original and has not been published elsewhere. The authors have no conflicts of interest to declare. All experimental protocols were reviewed and approved by the University of Alabama at Birmingham Institutional Animal Care and Use Committee.

## Data Availability

Cytokine densitometry data is deposited in Zenodo and is publicly available as of the date of publication (DOI: https://doi.org/10.5281/zenodo.15883027). No new code was generated or used in the analysis of this dataset. Any additional information required to reanalyze the data reported in this paper is available from the lead contact upon request.
